# Association between polymorphism within interleukin related genes and Graves' disease: a meta-analysis of 22 case-control studies

**DOI:** 10.18632/oncotarget.20114

**Published:** 2017-08-10

**Authors:** Yaqin Tu, Guorun Fan, Tianshu Zeng, Xiong Cai, Wen Kong

**Affiliations:** ^1^ Department of Otorhinolaryngology, Union Hospital, Tongji Medical College, Huazhong University of Science and Technology, Wuhan 430022, China; ^2^ Department of Endocrinology, Union Hospital, Tongji Medical College, Huazhong University of Science and Technology, Wuhan 430022, China; ^3^ Department of Hepatobiliary Surgery, Union Hospital, Tongji Medical College, Huazhong University of Science and Technology, Wuhan 430022, China

**Keywords:** Graves’ disease, interleukin, polymorphism, susceptibility, case/control study

## Abstract

Graves’ disease (GD) is a common autoimmune disorder with a genetic predisposition. There is strong evidence to suggest that both Th1 and Th2 circulating cytokines are involved in the development of GD. In this study, we conducted a meta-analysis to assess the impact of seven variations of five *IL*-related genes on the susceptibility to GD. A total of 22 case-control studies involving 5338 GD patients and 6446 healthy controls were included. The results showed that only one SNP rs1800795 in *IL*-6 was significantly associated with GD in homozygous model (CC vs. GG: OR = 2.714, 95% CI = 1.047–7.039, *p* = 0.04), heterozygous model (CG vs. GG: OR = 1.295, 95% CI = 1.013–1.655, *p* = 0.039), dominant model (CC+CG vs. GG: OR = 1.418, 95% CI = 1.122–1.793, *p* = 0.003) and additive model (C vs. G: OR = 1.432, 95% CI = 1.087–1.886, p = 0.011).To explain the heterogeneity, we performed the subgroup analysis by ethnicity. The ethnicity stratification revealed that the association between rs1800795 and GD tended to be much stronger for Asian than European population in homozygous, dominant, recessive, and additive models. The remaining 6 SNPs in 4 genes did not show any significant association with GD in any genetic models. Together, our data support that rs1800795 within the *IL*-6 gene confers genetic susceptibility for GD. Future large-scale studies are required to validate the associations between *IL*-6 and others *IL*-related genes and GD.

## INTRODUCTION

Graves’ disease (GD) is an autoimmune thyroid disease with a prevalence of 0.5% in the general population [1–2]. GD is characterized by the presence of thyroid-stimulating hormone (TSH) receptor antibodies, which leads to hyperthyroidism and goiter. The exact etiology of GD remains unknown; however, it is believed that genetic polymorphisms and environmental factors are both involved in the pathogenesis of GD. Large familial clustering and twin studies have proposed that about 79% of the risk for developing GD may be related to genetic factors, whereas 21% of them were related to the environmental and endogenous factors [3–4]. Genome-wide association studies (GWASs) have reported loci for GD on chromosomal region 5q31-q33 in Asian populations [5]. This region includes the T helper 1 (Th1) and Th2 gene cluster, which encodes certain cytokines, including the inflammation-associated proteins interleukin (IL)-4, IL-12, and IL-13. In addition, another GWAS revealed that IL-6 and IL-10 are related to the pathogenesis of GD [6–8].

IL-4, IL-6, IL-10, and IL-12 are produced by intra-thyroidal inflammatory cells and thyroid follicular cells. IL-13 is an important immunoregulatory cytokine involved in the IgE synthesis and is associated with Th2-mediated disease. The serum levels of IgE and IL-13 may be the indicators of remission or recurrence of GD [9–10]. Genetic factors that affect the induction or inhibition of these cytokines are the potential candidates for GD sensitivity. Indeed, emerging studies had shown that over 70 variations in 22 IL-related genes (*IL- 1B, IL-1α, IL-1β, IL-2, IL-3, IL-4, IL-5, IL-6, IL-8, IL-9, IL-10, IL-12, IL-12A, IL-13, IL-16, IL-17, IL-17F, IL-18, IL-18R, IL-21, IL-23, and IL-33*) are associated with GD. Nevertheless, the results are conflicting due to the limited sample size of each study. We, therefore, in the current report, conducted a meta-analysis of seven single nucleotide polymorphisms (SNPs) in five cytokine genes from all eligible case-control studies that included more than three studies to assess the associations among reported IL-related genes with GD.

## RESULTS

### Workflow for the identification of eligible datasets

**Figure 1:**
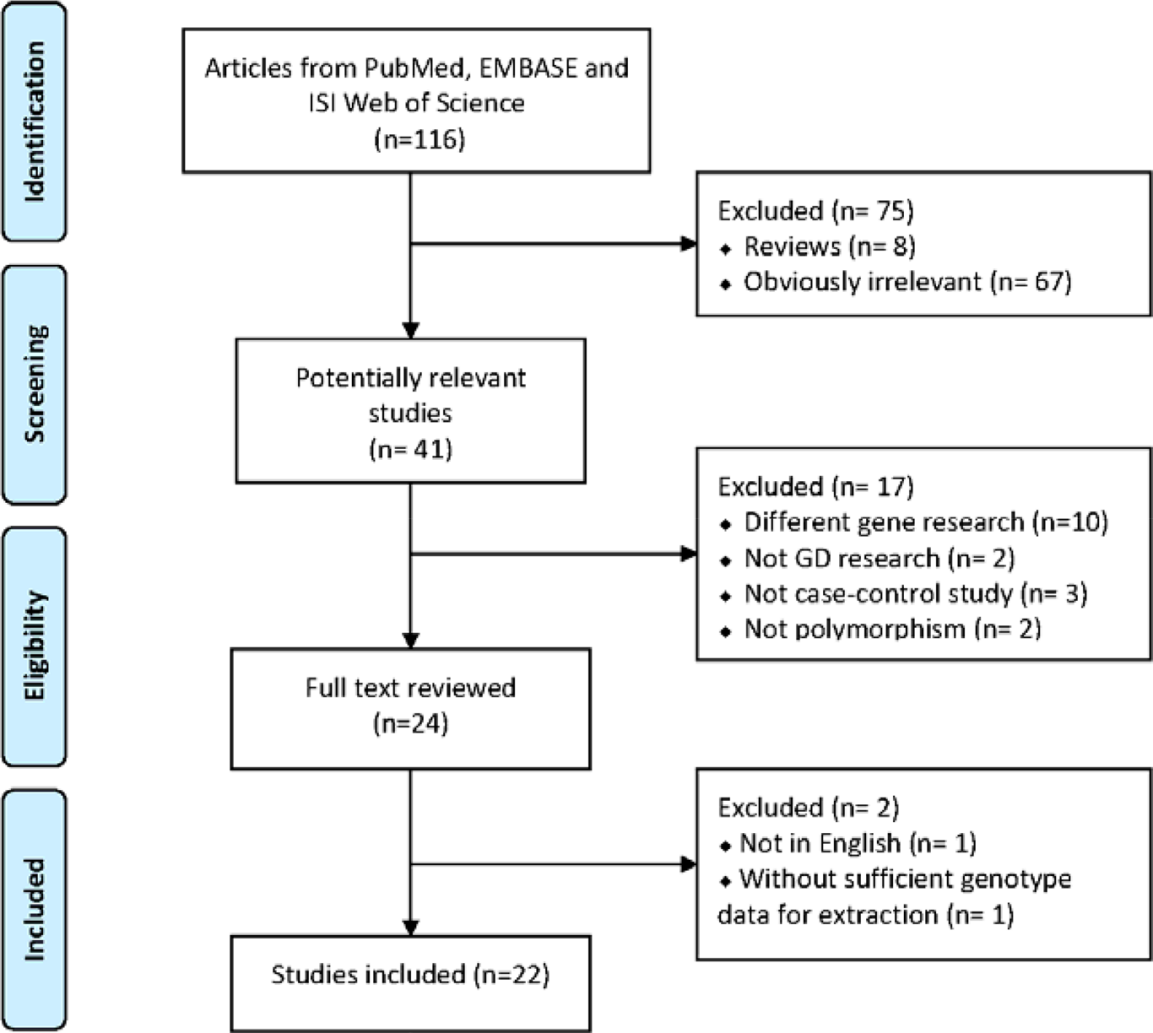
PRISMA flow diagram showing the search strategy

A total of 116 publications were characterized based on our keyword search. After screening the titles and abstracts, 67 studies were identified as irrelevant, and eight articles were characterized as reviews. Additionally, 17 studies were excluded because 10 of the articles focused on different genes. Another seven articles were excluded because they were not on GD research (two studies), were not case control studies (three studies), or did not assess polymorphisms (two articles). Among the remaining 24 publications, two studies were also rejected as they either failed to provide detailed genotyping information (one article) or were published in non-English journals (one study) (Figure 1).

### Characteristics of the selected datasets

A total of 22 case control datasets were identified based on our inclusion criteria. Of these, 22 studies, including 5338 patients with GD and 6446 healthy controls, were analyzed. The principal characteristics and genotype distributions of the identified studies are shown in Table 1. Among the included articles, 16 studies were from Asian populations [6–7, 9, 11–23] and 6 studies were from European populations [24–29]. Genotypic distribution for all SNPs in controls was in consistent with HWE (*p* > 0.05) except for the 3 datasets highlighted in bold (Table 1). The quality of each study was assessed through Newcastle-Ottawa assessment scale (NOS), as shown in Table 2. All included studies scored 7 or 8, indicating sufficient quality for their inclusion in the meta-analysis.

**Table 1:** Characteristic of datasets included for meta-analysis

ID	Author	Year	Ethnicity	Genotyping method	Study design	Gene	Case/Control	SNP loci	GD			Control			*pHWE*
									11	12	22	11	12	22	
1	Jung-Pil Jang	2016	Asian	hybridization	CC	IL-13	60/192	rs1800925	36	24	0	129	61	2	0.07
2	Duraes	2014	European	Taqman	CC	IL-6	111/735	rs1800795	13	61	37	92	324	319	0.5
3	Faruk Kutluturk	2013	European	PCR-SSP	CC	IL-6	100/124	rs1800795	12	36	52	6	41	77	0.86
4	Yann-Jinn Lee	2011	Asian	Taqman	CC	IL-4	220/904	rs2243250	9	64	147	38	255	611	0.087
5	N. Inoue	2011	Asian	PCR-RFLP	CC	IL-13	78/68	rs1800925	60	16	2	53	14	1	0.95
6	Nan Liu	2011	Asian	GenomeLab SNPstream	CC	IL-10	725/696	rs1800872	321	326	78	299	310	87	0.63
7	Omid Khalilzadeh	2010	Asian	PCR-SSP	CC	IL-4	107/139	rs2243250	50	52	5	10	129	0	**0.00**
							107/139	rs2070874	29	50	28	61	78	0	**0.00**
8	W, Zhu	2010	Asian	GenomeLab SNPstream	CC	IL-4	731/716	rs2070874	453	253	25	461	231	24	0.45
9	Mehdi Anvari	2010	Asian	PCR-SSP	CC	IL-6	107/139	rs1800795	27	63	17	4	93	42	**0.00**
						IL-12	107/140	rs3212227	34	53	20	72	60	8	0.32
10	Kelvin K. L. Chong	2008	Asian	PCR-RFLP	CC	IL-13	177/151	rs1800925	120	51	6	106	44	1	0.11
								rs20541	68	81	28	66	67	18	0.87
11	Nanba	2008	Asian	Sequencing	CC	IL-4	50/26	rs2243250	4	22	24	2	11	13	0.88
12	Rong-Hsing Chen	2007	Asian	GenomeLab SNPstream	CC	IL-4	104/105	rs2243250	4	32	68	2	36	67	0.25
13	Ming-Yuh Shiau	2007	Asian	PCR-RFLP	CC	IL-10	133/134	rs1800872	62	54	17	72	52	10	0.89
						IL-4	130/101	rs2070874	93	34	3	68	31	2	0.47
							137/119	rs2243250	6	48	83	6	35	78	0.43
14	Yuji Hiromatsu	2006	Asian	Sequencing	CC	IL-12	329/226	rs3212227	77	162	90	39	120	67	0.24
15	Yang	2005	Asian	Sequencing	CC	IL-4	187/131	rs2243250	7	51	129	2	46	83	0.12
16	Yukio Ikeda	2004	Asian	PCR-RFLP	CC	IL-12	90/123	rs3212227	22	39	29	29	64	30	0.65
17	Yuji Hiromatsu	2004	Asian	PCR-RFLP	CC	IL-13	310/244	rs1800925	219	83	8	143	88	13	0.91
								rs20541	166	123	21	113	100	31	0.24
18	Bednarczuk, T	2004	Asian	PCR-RFLP	CC	IL-6	279/186	rs1800795	56	138	85	27	101	58	0.11
19	Karen F, Tait	2004	European	Invader assay	CC	IL-10	630/846	rs1800872	32	234	364	35	290	521	0.50
20	Tomasz Bednarczuk	2003	European	SSCP	CC	IL-13	261/168	rs1800925	127	108	26	89	63	16	0.33
								rs20541	148	94	19	94	66	8	0.40
21	Heward	2001	European	PCR-RFLP	CC	IL-4	381/285	rs2243250	277	99	5	222	58	5	0.59
22	Hunt	2000	European	PCR-SSP	CC	IL-4	138/101	rs2243250	122	15	1	75	23	3	0.46

11: wild-type homozygote, 12: heterozygote, 22: variant homozygote.

CC: case/control.

HWE: Hardy-Weinberg equilibrium.

PCR-SSCP: PCR-single-strand conformation polymorphism.

PCR-SSP: PCR-sequence specific primer.

**Table 2:** Quality assessments of case-control studies according to the Newcastle Ottawa Scale

Study ID	Author	Year	Selection				Comparability		Exposure			Total score
			a	b	c	d	e	f	g	h	i	
1	Jung-Pil Jang	2016	^*^	^*^	/	/	^*^	^*^	^*^	^*^	^*^	7
2	Duraes	2014	^*^	^*^	/	^*^	^*^	^*^	^*^	^*^	^*^	8
3	Faruk Kutluturk	2013	^*^	^*^	/	^*^	^*^	^*^	^*^	^*^	^*^	8
4	Yann-Jinn Lee	2011	^*^	^*^	/	^*^	^*^	^*^	^*^	^*^	^*^	8
5	N. Inoue	2011	^*^	^*^	/	/	^*^	^*^	^*^	^*^	^*^	7
6	Nan Liu	2011	^*^	^*^	/	^*^	^*^	^*^	^*^	^*^	^*^	8
7	Omid Khalilzadeh	2010	^*^	^*^	/	^*^	^*^	/	^*^	^*^	^*^	7
8	W, Zhu	2010	^*^	^*^	/	^*^	^*^	^*^	^*^	^*^	^*^	8
9	Mehdi Anvari	2010	^*^	^*^	/	^*^	^*^	/	^*^	^*^	^*^	7
10	Kelvin K. L. Chong	2008	^*^	^*^	/	^*^	^*^	^*^	^*^	^*^	^*^	8
11	Nanba	2008	^*^	^*^	/	/	^*^	^*^	^*^	^*^	^*^	7
12	Rong-Hsing Chen	2007	^*^	^*^	/	^*^	^*^	^*^	^*^	^*^	^*^	8
13	Ming-Yuh Shiau	2007	^*^	^*^	/	/	^*^	^*^	^*^	^*^	^*^	7
14	Yuji Hiromatsu	2006	^*^	^*^	/	^*^	^*^	^*^	^*^	^*^	^*^	8
15	Yang	2005	^*^	^*^	/	^*^	^*^	/	^*^	^*^	^*^	
16	Yukio Ikeda	2004	^*^	^*^	/	^*^	^*^	^*^	^*^	^*^	^*^	8
17	Yuji Hiromatsu	2004	^*^	^*^	/	^*^	^*^	^*^	^*^	^*^	^*^	8
18	Bednarczuk, T	2004	^*^	^*^	/	/	^*^	^*^	^*^	^*^	^*^	7
19	Karen F, Tait	2004	^*^	^*^	/	^*^	^*^	/	^*^	^*^	^*^	7
20	Tomasz Bednarczuk	2003	^*^	^*^	/	/	^*^	^*^	^*^	^*^	^*^	7
21	Heward	2001	^*^	^*^	/	^*^	^*^	/	^*^	^*^	^*^	7
22	Hunt	2000	^*^	^*^	/	^*^	^*^	/	^*^	^*^	^*^	7

Publication quality check list:

a: Is the case definition adequate?;

b: Representativeness of the cases;

c: Selection of Controls;

d: Definition of Controls;

e: study controls for ethnicity;

f: study controls for any additional factor;

g: Ascertainment of exposure;

h: Same method of ascertainment for cases and controls;

i: Non-Response rate.

### Association between IL-related gene polymorphism and Graves’ disease

**Figure 2:**
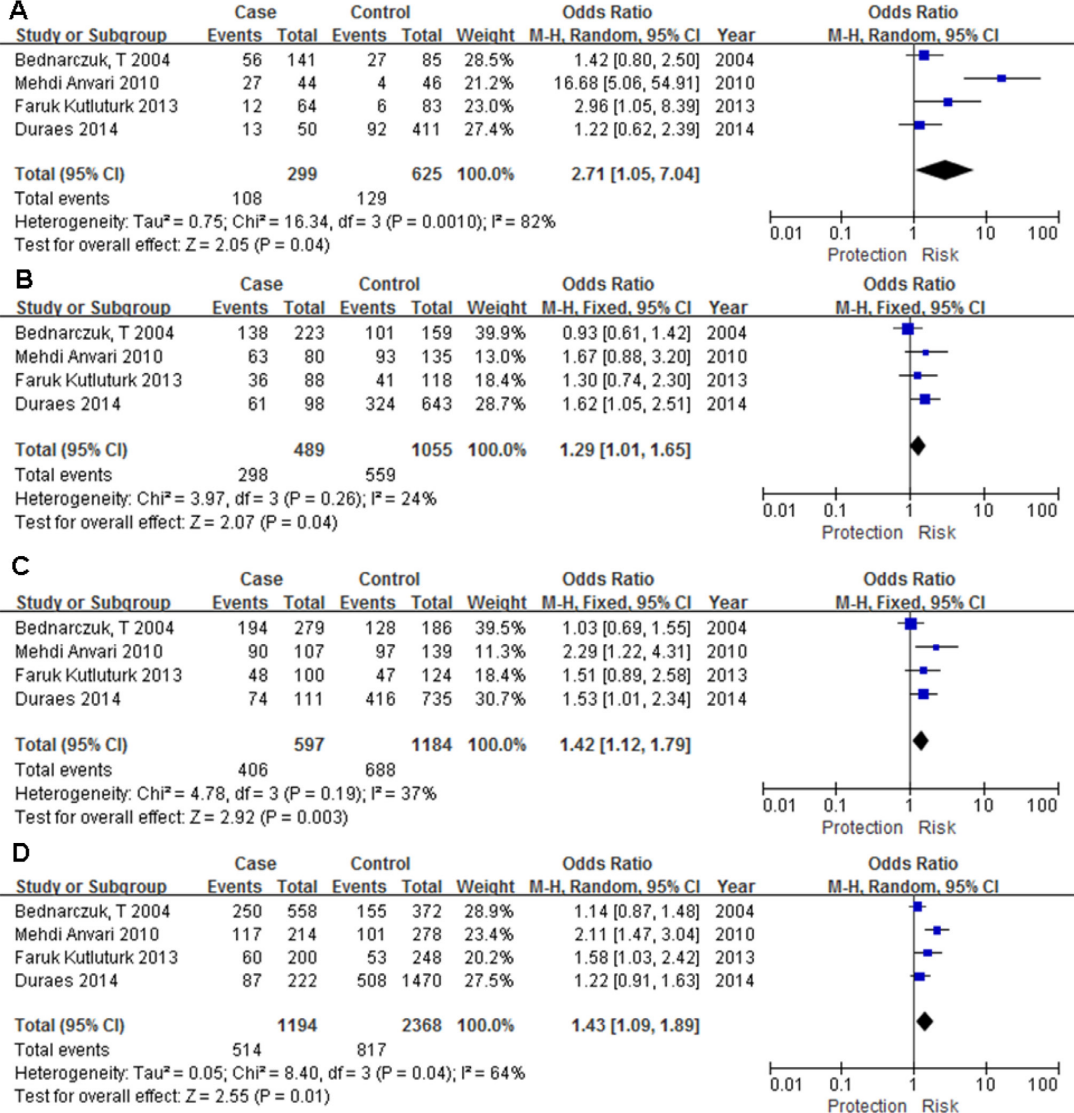
Forest plot for the association between *IL-6* rs1800795 polymorphism and Graves’ disease (**A**) homozygous model (CC vs. GG), (**B**) heterozygous model (CG vs. GG), (**C**) dominant model (CC+CG vs. GG), (**D**) additive model (C vs. G).

We performed a meta-analysis of seven SNPs in five *IL* -related genes, including *IL-4* (rs2243250 and rs2070874), *IL-6* (rs1800795), *IL-10* (rs1800872), *IL-12* (rs3212227), and *IL-13* (rs1800925 and rs20541). The number of the included datasets on each SNP ranged from 3 to 8. Unexpectedly, only one SNP (rs1800795) in *IL-6* was found to have a significant association with GD in the homozygous model (CC vs. GG: OR = 2.714, 95% CI = 1.047 - 7.039, *p* = 0.04), heterozygous model (CG vs. GG: OR = 1.295, 95% CI = 1.013 - 1.655, *p* = 0.039), dominant model (CC+CG vs. GG: OR = 1.418, 95% CI = 1.122 - 1.793, *p* = 0.003) and additive model(C vs. G: OR = 1.432, 95% CI = 1.087 - 1.886, *p* = 0.011) (Table 2; Figure 2). Our meta-analysis of rs1800795 was hampered by high heterogeneity. A random effect model was thus used. To explain the heterogeneity, we performed the subgroup analysis by ethnicity. The ethnicity stratification revealed that the association between rs1800795 and GD tended to be much stronger for Asian populations than for European populations in the homozygous, dominant, recessive, and additive models (Supplementary Figure 1).

The remaining 6 SNPs in the 4 genes did not show significant association with GD in any genetic model (Table 3). Among the insignificant polymorphisms, rs2243250 in *IL-4* (I^2^ < 85.2%), rs2070874 in *IL-4* (I^2^ < 88.8%), rs1800872 in *IL-10* (I^2^ < 53.3%), rs3212227 in *IL-12* (I^2^ < 87.0%), rs1800925 in *IL-13* (I^2^ < 63.9%), and rs20541 in *IL-13* (I^2^ < 75.9%) were hampered by high heterogeneity. Considering the limited number of studies, we only performed subgroup analysis of variations with more than 3 articles (rs2243250 in *IL-4* and rs1800925 in *IL-13*). Ethnicity stratification showed that these two SNPs did not show significant associations with GD in Asian and European populations (Supplementary Table 1).

**Table 3:** Results for meta-analysis of polymorphism within Interleukin related genes with Graves’ disease risk

Gene	Polymorphism	No. of datasets	Ethnicity	Genetic model	OR(95% CI)	*p* value	Test of heterogeneity		*p* for publication bias^a^
							I^2^	*p* value	
IL-4	rs2243250	8	Asian, European	CC vs. TT	1.225 (0.777, 1.931)	0.382	0%	0.86	0.308
				CT vs. TT	0.959 (0.776, 1.184)	0.695	21.8%	0.256	0.629
				CC+CT vs. TT	0.988 (0.806, 1.211)	0.905	0%	0.431	0.872
				CC vs. CT+TT	1.800 (0.814, 3.981)	0.147	85.2%	0.000	0.378
				C vs. T	1.188 (0.906, 1.557)	0.213	69.8%	0.002	0.392
IL-4	rs2070874	3	Asian, European	CC vs. TT	0.261 (0.02,3.366)	0.303	86.2%	0.001	0.451
				CT vs. TT	0.273 (0.022,3.4710	0.317	85.7%	0.001	0.386
				CC+CT vs. TT	0.269 (0.02,3.551)	0.319	86.6%	0.001	0.430
				CC vs. CT+TT	0.871 (0.526,1.27)	0.369	68.0%	0.044	0.810
				C vs. T	0.747 (0.416,1.342)	0.330	88.8%	0.000	0.763
IL-6	rs1800795	4	Asian, European	CC vs. GG	2.714 (1.047, 7.039)	0.04	81.6%	0.01	0.117
				CG vs. GG	1.295 (1.013, 1.655)	0.039	24.4%	0.265	0.541
				CC+CG vs. GG	1.418 (1.122, 1.793)	0.003	37.2%	0.189	0.178
				CC vs. CG+GG	2.305 (0.947, 5.613)	0.066	82.1%	0.001	0.221
				C vs. G	1.432 (1.087, 1.886)	0.011	64.3%	0.038	0.228
IL-10	rs1800872	3	Asian, European	AA vs. CC	1.123 (0.86,1.467)	0.394	48.1%	0.146	0.388
				AC vs. CC	1.127 (0.942,1.35)	0.191	40%	0.366	0.299
				AA+AC vs. CC	1.135 (0.955,1.348)	0.15	36.3%	0.208	0.280
				AA vs. AC+CC	1.028 (0.86,1.23)	0.761	10.8%	0.326	0.816
				A vs. C	1.065 (0.953,1.19)	0.265	53.3%	0.117	0.276
IL-12	rs3212227	3	Asian	CC vs. AA	0.634 (0.206,1.949)	0.427	86.7%	0.001	0.2114
				CA vs. AA	0.677 (0.384,1.193)	0.177	59.8%	0.083	0.039
				CC+CA vs. AA	0.636 (0.3,1.348)	0.237	79.3%	0.008	0.095
				CC vs. CA+A	0.883 (0.417,1.869)	0.744	83.7%	0.002	0.729
				C vs. A	0.804 (0.473,1.367)	0.421	87.0%	0.000	0.388
IL-13	rs1800925	5	Asian, European	TT vs. CC	1.056 (0.652,1.712)	0.824	38%	0.164	0.595
				TC vs. CC	1.018 (0.616,1.68)	0.946	0%	0.489	0.471
				TT+TC vs. CC	1.055 (0.656,1694)	0.826	23.8%	0.263	0.556
				TT vs. TC+CC	1.009 (0.712,1.429)	0.961	61.1%	0.036	0.363
				T vs. C	1.005 (0.739,1.367)	0.974	63.9%	0.026	0.438
IL-13	rs20541	3	Asian, European	AA vs. GG	1.014 (0.44,2.337)	0.973	75.9%	0.016	0.467
				AG vs. GG	0.991 (0.504,1.949)	0.978	62.7%	0.068	0.406
				AA+AG vs. GG	1.006 (0.473,2.14)	0.987	72.9%	0.025	0.499
				AA vs. AG+GG	1.083 (0.869,1.35)	0.476	39.7%	0.19	0.005
				A vs. G	1.036 (0.756,1.418)	0.828	70%	0.036	0.146

^a^:Egger’s test was performed to assess publication bias.

### Publication bias

**Figure 3:**
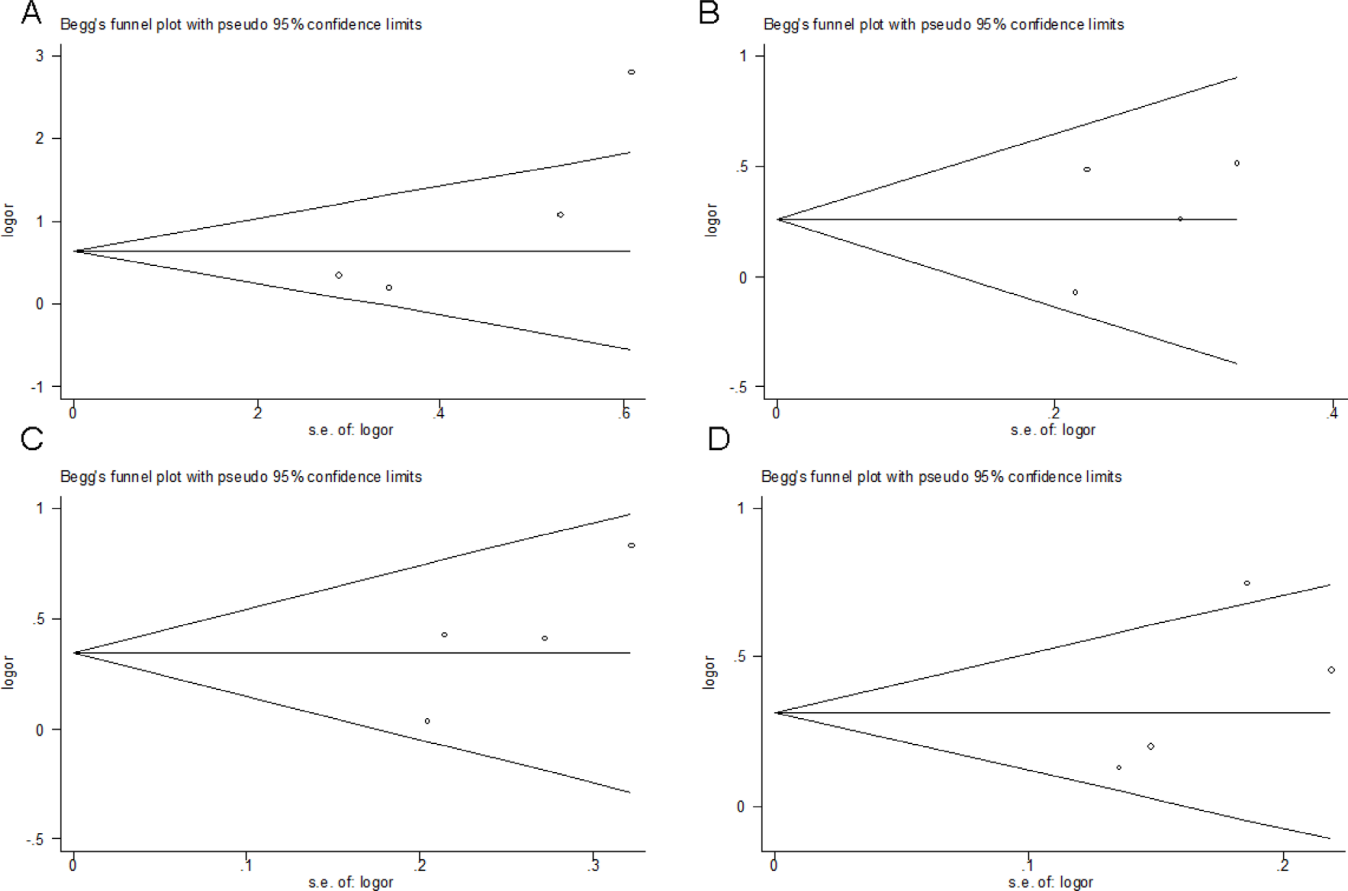
Funnel plot analysis to detect publication bias (SNP rs1800795 in *IL-6*) Each point represents a separate study for the indicated association. (**A**) homozygous model (CC vs. GG), (**B**) heterozygous model (CG vs. GG), (**C**) dominant model (CC+CG vs. GG), (**D**) additive model (C vs. G).

Begg's funnel plot and Egger's test were performed to assess the publication bias. The shape of the funnel plots appeared to be symmetrical (SNP rs1800795 in *IL-6*) and the Egger's test did not show any evidence of publication bias (Figure 3). The sensitivity analyses also indicated that results of our study were stable and reliable (data not shown).

## DISCUSSION

In the present study, we examined the association between variations in IL-related genes and GD. The results of our overall meta-analysis supported that only G->C mutation at rs1800795 in IL-6 was a risk factor for GD. The other 6 variations in the 4 genes (IL-4 rs2243250, IL-4 rs2070874, IL-10 rs1800872, IL-12 rs3212227, IL-13 rs1800925 and IL-13 rs20541) did not show a significant association with GD in any genetic model. Considering the fact that the ethnic background may affect the results of genetic association studies, we performed a subgroup analysis by ethnicity and found that the association was more apparent among Asian populations, indicating that IL-6 polymorphism may exert varying effects in different populations. This is perhaps because different populations are exposed to diverse environments during their evolution and different life styles. These results were consistent with the findings of Mehdi Anvari *et al* in an Iranian population [14]. IL-6 plays an important role in the growth and differentiation of lymphocytes, which might contribute to the promotion of thyroid receptor antibody synthesis during the course of GD. IL-6 rs1800795, which is located in the 5′ promoter region at position -174(G-174C), appears to influence IL-6 production. Moreover, there are evidences indicating that the C allele results in lower transcriptional activity than the G allele [30–31]. In line with this notion, subjects carrying the putative risk C/C genotype show lower plasma IL-6 levels than subjects carrying the G/G genotype. These findings suggest that the IL6 rs1800795 polymorphism is functional and may be related to the pathogenesis of GD.

Except for IL-6, six variations in these genes (*IL-4* rs2243250, *IL-4* rs2070874, *IL-10* rs1800872, *IL-12* rs3212227, *IL-13* rs1800925, and *IL-13* rs20541) did not show significant associations with GD in our meta-analysis. IL-10 is a major anti-inflammatory cytokine that is secreted by activated T cells, B cells, monocytes, and thymocytes [32]. *IL-10* rs1800872 showed no association with GD in all of the tested population and had no heterogeneity. Thus, it is not likely to be a genetic marker for GD. Another five insignificant SNPs (*IL-4* rs2243250, *IL-4* rs2070874, *IL-12* rs3212227, *IL-13* rs1800925, and *IL-13* rs20541) also did not show an association with GD in all of the tested population with mild to high heterogeneities. IL-4 is a member of Th2 cytokines with anti-inflammatory properties, which reduces the production of proinflammatory cytokines and destructive enzymes by monocytes. Interestingly, we found that there were inconsistent conclusions even within the same ethnic group. For example Nanba and colleagues showed that rs2243250 in *IL-4* was significantly associated with GD in the Japanese population [16], whereas Lee and colleagues failed to detect an association between *IL-4* polymorphisms (rs2243250) and GD risk in a Chinese dataset [13]. This discrepancy is likely due to the differences in their lifestyle and diet, social and emotional stress, as well as their medical care and economic conditions. Of course, other factors such as study design and limited sample size, may also contribute to such discrepancies. Given that the associations between these genes and GD are still controversial, further replication studies of *IL-4* gene among different population are warranted. IL-13 is an important immunoregulatory protein produced primarily by activated Th2 cells and is involved in B cell maturation. High serum IL-13 levels have been observed in patients with GD, which decrease significantly after treatment, suggesting that IL-13 gene polymorphisms are contributing to the development and severity of GD. However, we did not detect association of IL-13 with GD in our meta-analysis. This may be due to the limited number of included studies. IL-12 is an important cytokine that regulates innate resistance and adaptive immunity [33–34]. It has been demonstrated that the imbalance between Th1 and Th2 cytokine production is highly correlated with the induction and development of several autoimmune diseases including GD. Although rs3212227 in *IL-12* are not significant in our meta-analysis, another SNP in IL-12 (rs568408) had show significant association with GD [35]. For this reason, the contributing role of the genetic variants of IL-12 needs further conformation in specific population.

Our meta-analysis has some key advantages. First, although other authors published such meta-analysis, they only studied the association between one of the interleukin genes and GD. However, our meta-analysis evaluated the “functional synergies” of Th1 (IL-12) and Th2 (IL-4, IL-6, IL-10 and IL-13) cytokines in autoimmune inflammation in the GD. Second, to guarantee the quality of this study, we included the most updated literature and used explicit criteria for study inclusion and a strict procedure for data extraction. Third, a substantial number of subjects were pooled from individual studies, which significantly increased the statistical power. However, there are several limitations in our study. First, the controls were hospital-based study in our included literatures. Compared with hospital-based study, a population-based case-control study can reduce more selection bias and have higher confidence. Second, our search was restricted to English-language studies. Some potential studies which were published in other languages or unpublished have been systematically excluded. This may explain some publication bias in our meta-analysis, which may have affected the results of this meta-analysis in as far as those studies that had produced negative results might not have been published. Third, the small number of published studies may lead to erroneous conclusions, especially among different ethnic group. What's more, since we were not able to obtain the original data, our further evaluation of gene-to-environment interactions was limited.

In summary, the results of our meta-analysis identified that only rs1800795 within the *IL-6* gene is associated with increased risk for developing GD. Owing to the limited number of included studies, future studies with a large dataset focusing on address their functional relevance would be necessary for fully establishing their effect on GD susceptibility.

## MATERIALS AND METHODS

### Eligible studies

PubMed, EMBASE and ISI Web of Science were searched (the last search was conducted on March 1, 2017) using the following search terms: “ILs” or “interleukins” and “Graves’ disease” and “polymorphism”. Reference, which were listed in each identified article, were also searched manually to identify additional eligible studies.

### Validity assessment

To be eligible, the following inclusion criteria were established: (1) a human case-control study of a polymorphism associated with Graves’ disease; (2) studies that included sufficient genotype data for extraction. Main exclusion criteria of studies were as follows: (1) case reports, letters, reviews, and editorial articles; (2) literature not containing information regarding diabetes research; (3) study involving only a case population; and (4) study not written in English. In the case of multiple studies by the same researchers involving the same or overlapping data sets, we selected the most recent study with the largest number of participants.

### Data extraction and quality assessment

Two curators (Yaqin Tu and Guorun Fan) independently extracted information from included studies. Disagreement was resolved by discussion between the two authors. The following data were extracted: first author's name, year of publication, the ethnicities of the individuals involved, the genotyping method, number of cases and controls for each genotype, and the Hardy-Weinberg equilibrium (HWE) among the controls. Ethnicity was categorized as East Asian and European. A double-check procedure was performed to ensure accuracy of data entry. To evaluate the quality of included studies, we adopted the Newcastle-Ottawa Scale (NOS) with a nine-star system; this scale assesses the quality of cohort and case-control studies. NOS focuses on three separate sections of stars represents the assessment score. The maximal score of NOS is 9 stars: 4 stars for the selection process, 2 stars for comparability, and 3 stars for exposure/outcome. A score of seven and above was represents the high-quality of study.

### Statistical analysis

The strength of associations between IL-related genes and Graves’ disease risk was measured by ORs with 95% CIs. We explored the association between IL-related genes and Graves’ disease in homozygote (XX vs. xx), heterozygote (Xx vs. xx), dominant (Xx + XX vs. xx), recessive (XX vs. Xx + xx), and additive (X vs. x) models respectively. Hardy–Weinberg equilibrium (HWE) was evaluated by the goodness-of-fit *χ2* test for genotypes in the control group. Chi-squared-based *Q*-statistic test was employed to assess the between-study heterogeneity, and in any case *p* < 0.10 was considered with significant heterogeneity between datasets. Once the effects were assumed to be homogenous, fixed-effects model was then applied (the Mantel-Haenszel method); otherwise, the random-effects model (DerSimonian and Laird method) was employed appropriately. Sensitivity analysis was performed to assess the influence of each individual study by omitting 1 study at a time and calculating a pooled estimate for the remainder of the studies. The inverted funnel plots and Egger's regression test were used to investigate publication bias. Potential publication bias was assessed with funnel plots of the effect sizes versus the standard errors; Begg's test was used to identify significant asymmetry. If there is evidence of publication bias, funnel plot is noticeably asymmetric. Concerning the significance level of the Begg's and Egger's tests was set at 0.05. All statistical tests carried out in the present report were two tailed. All analyses were conducted using the Review Manager 5.0.23 (Cochrane Library Software, Oxford, UK) and STATA 11.0 software (STATA Corporation, College Station, TX, USA).

## SUPPLEMENTARY MATERIALS FIGURE AND TABLE



## Abbreviations

GD: Graves’ disease
IL: Interleukin
Th1: T helper 1
TSH: thyroid-stimulating hormone
GWAS: genome-wide association studies
NOS: Newcastle-Ottawa Scale
CI: confidence interval
OR: odds ratio
SNPs: single nucleotide polymorphisms
HWE: Hardy-Weinberg equilibrium
