# Supplementary material for: Association between polymorphism within interleukin related genes and Graves' disease: a meta-analysis of 22 case-control studies
Source: Oncotarget. 2017 Aug 10;8(58):98993–9002. doi: 10.18632/oncotarget.20114 (PMC5716784; doi:10.18632/oncotarget.20114)
Supplement: Supplementary file 1 [file oncotarget-08-98993-s001.pdf]

# Association between polymorphism within interleukin related genes and Graves' disease: a meta-analysis of 22 case-control studies

## SUPPLEMENTARY MATERIALS

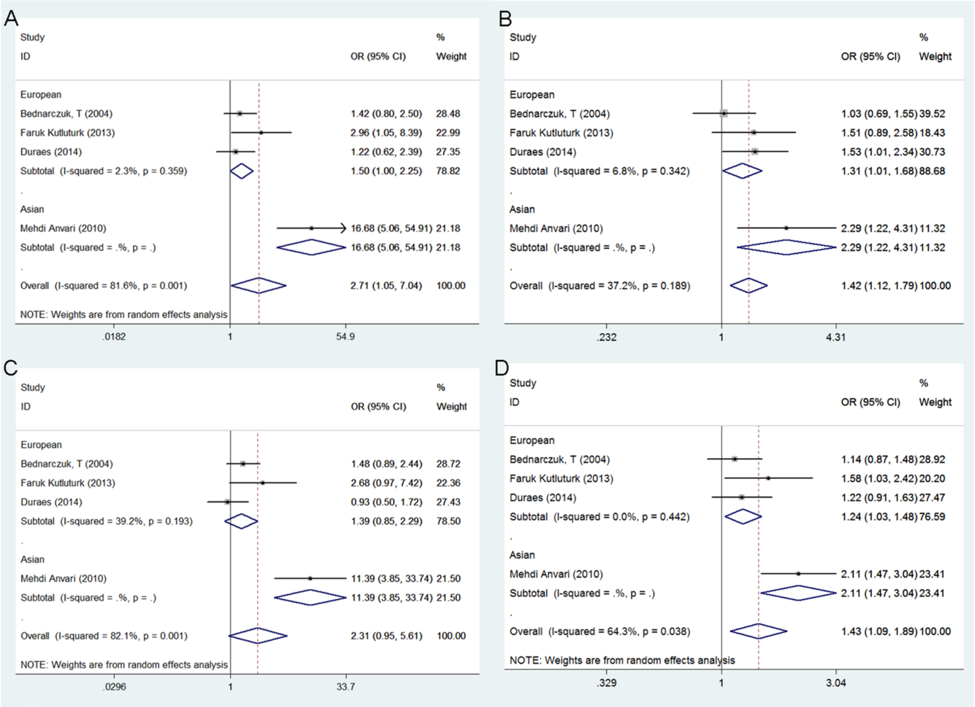


**Supplementary Figure 1: Forest plot for the association between IL-6 rs1800795 polymorphism and Graves' disease after ethnicity stratification.** (**A**) homozygous model (CC vs. GG), (**B**) dominant model (CC+CG vs. GG), (**C**) recessive model (CC vs. CG+GG), (**D**) additive model (C vs. G).

## Supplementary Table 1: Subgroup analysis of rs2243250 in *IL-4* and rs1800925 in *IL-13*

| Gene | Polymorphism | Genetic model | Ethnicity | No. of datasets | OR(95% CI) | *p* value | Test of heterogeneity | |
|---|---|---|---|---|---|---|---|---|
| | | | | | | | $I^2$ | *p* value |
| IL-4 | rs2243250 | CC vs. TT | Asian | 6 | 1.124 (0.677, 1.866) | 0.652 | 0% | 0.885 |
| | | | European | 2 | 1.778 (0.615, 5.142) | 0.288 | 5.6% | 0.303 |
| | | CT vs. TT | Asian | 6 | 0.937 (0.755, 1.162) | 0.554 | 36.5% | 0.163 |
| | | | European | 2 | 1.767 (0.574, 5.436) | 0.321 | 0% | 0.92 |
| | | CC+CT vs. TT | Asian | 6 | 0.965 (0.784, 1.188) | 0.736 | 4.4% | 0.388 |
| | | | European | 2 | 1.808 (0.623, 5.249) | 0.276 | 0.0% | 0.389 |
| | | CC vs. CT+TT | Asian | 6 | 2.003 (0.683, 5.879) | 0.206 | 81.0% | 0.000 |
| | | | European | 2 | 1.364 (0.401, 4.644) | 0.619 | 90.0% | 0.002 |
| | | C vs. T | Asian | 6 | 1.166 (0.888, 1.532) | 0.268 | 60.5% | 0.027 |
| | | | European | 2 | 1.393 (0.454, 4.277) | 0.563 | 90.1% | 0.001 |
| IL-13 | rs1800925 | TT vs. CC | Asian | 2 | 0.424 (0.102, 1.751) | 0.236 | 0% | 0.545 |
| | | | European | 2 | 1.413 (0.511, 3.908) | 0.505 | 69.3% | 0.071 |
| | | TC vs. CC | Asian | 2 | 0.463 (0.109, 1.959) | 0.295 | 0% | 0.465 |
| | | | European | 2 | 1.206 (0.691, 2.106) | 0.510 | 0% | 0.529 |
| | | TT+TC vs. CC | Asian | 2 | 0.436 (0.106, 1.798) | 0.251 | 0% | 0.514 |
| | | | European | 2 | 1.338 (0.614, 2.913) | 0.464 | 50.2% | 0.156 |
| | | TT vs. TC+CC | Asian | 2 | 0.848 (0.608, 1.185) | 0.334 | 0% | 0.839 |
| | | | European | 2 | 1.202 (0.603, 2.394) | 0.601 | 85.5% | 0.009 |
| | | T vs. C | Asian | 2 | 0.838 (0.624, 1.125) | 0.240 | 0% | 0.97 |
| | | | European | 2 | 1.196 (0.673, 2.127) | 0.541 | 86.3% | 0.007 |
